# Supplementary material for: Mammalian Homologue NME3 of DYNAMO1 Regulates Peroxisome Division
Source: Int J Mol Sci. 2020 Oct 28;21(21):8040. doi: 10.3390/ijms21218040 (PMC7662248; doi:10.3390/ijms21218040)
Supplement: Supplementary file 1 [file ijms-21-08040-s001.pdf]

Supplementary Materials.

Mammalian homologue NME3 of DYNAMO1 regulates peroxisome division

Masanori Honsho, Yuichi Abe, Yuuta Imoto, Zee-Fen Chang, Hanna Mandel, Tzipora C. Falik-Zaccai and Yukio Fujiki

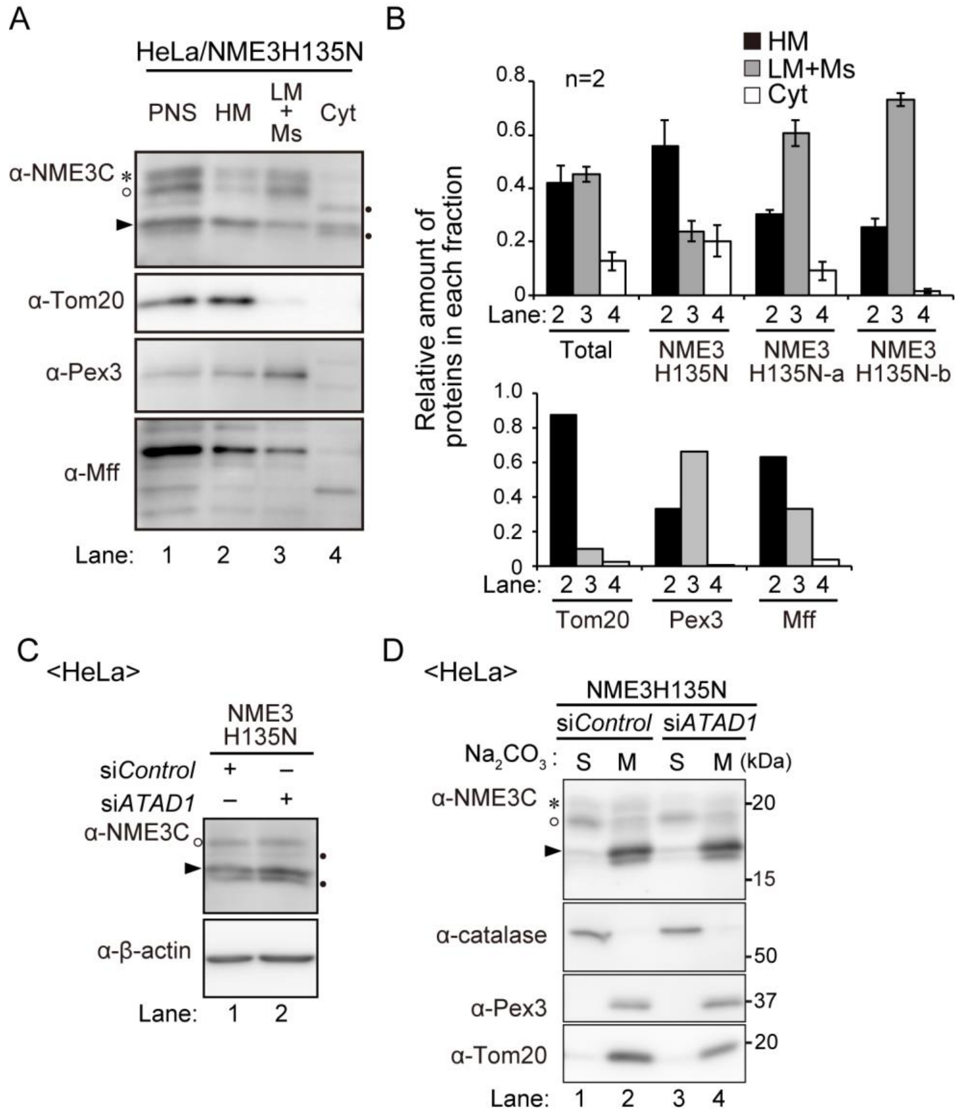

Supplementary Figure S1. Intracellular distribution of NME3H135N and its membrane integrity. *A*, distribution of NME3H135N was assessed as described in Fig. 8C. Three bands, NME3H135N, NME3H135N-a and NME3H135N-b were marked by solid arrowhead, asterisk, and open circle, respectively. Dots, non-specific bands. B, subcellular distribution of NME3H135N and marker proteins was represented in each fraction by taking as 1 total amount of respective proteins detected in lanes 2-4 (n = 2). Solid, gray, and open bars indicate the levels in HM, LM plus Ms, and cytosol fractions, respectively. The values were represented as means ± ranges of two independent experiments (upper panel) and a single experiment (lower panel), respectively. NME3H135N was mostly in HM (solid bar) fraction and partly in both post-HM (gray bar) and cytosolic fractions (open bar), whereas NME3H135N-a and NME3H135N-b were mainly recovered in the post-HM fraction. *C*, NME3H135N level is increased by ATAD1 knockdown. Transfection of *siControl* (lane 1) and *siATAD1* (lane 2) to HeLa cells expressing NME3H135N was performed as in Fig. 7J. Two bands, NME3H135N and NME3H135N-b, marked by solid arrowhead and open circle were detected. β-actin, a loading control. Dots indicate non-specific bands. *D*, organelle fractions (100,000 x *g* pellet fraction of PNS) each from the HeLa cells described in *C* were treated with 0.1 M Na_2CO_3 and separated into soluble (S) and membrane (M) fractions as in Fig. 8E. NME3H135N was recovered in the membrane fraction, while both NME3H135N-a and NME3H135N-b were recovered in both membrane and soluble fractions.
